# Factors Affecting the Public Intention to Repeat the COVID-19 Vaccination: Implications for Vaccine Communication

**DOI:** 10.3390/healthcare11091235

**Published:** 2023-04-26

**Authors:** Yubin Lee, Kunhee Park, Jeonghoon Shin, Jeonghyeon Oh, Yeongeun Jang, Myoungsoon You

**Affiliations:** 1 Department of Public Health, Graduate School of Public Health, Seoul National University, Seoul 08826, Republic of Korea; ubeanee@snu.ac.kr (Y.L.); jayshin@snu.ac.kr (J.S.); 2 Gyeonggi Infectious Disease Control Center, Suwon 16508, Republic of Korea; kpark.ghealth@gmail.com (K.P.); jhyeon.paz@gmail.com (J.O.); yeplus@gg.go.kr (Y.J.)

**Keywords:** COVID-19, SARS-CoV-2, vaccination intention, vaccine communication, hierarchical logistic regression

## Abstract

Although most of the pandemic-related mandatory restrictions have been lifted or eased, vaccination is still recommended as an effective measure to minimize the damage from COVID-19 infection. Since COVID-19 eradication is unlikely, it is necessary to understand the factors affecting the public’s vaccination intention when COVID-19 vaccination is continuously recommended. This study aims to explore the factors that affect the intention to repeat the COVID-19 vaccination in South Korea. An online survey was conducted in January 2022 with adults living in Gyeonggi-do, South Korea. In a hierarchical logistic regression analysis, sociodemographic factors, COVID-19 infection-related factors, COVID-19 vaccination-related factors, sociocultural factors, and communication factors were taken into account. In this study, more than three-quarters (78.1%) of Koreans were willing to repeat the COVID-19 vaccination. People who had high-risk perceptions, had been vaccinated against COVID-19 at least once, had more authoritarian attitudes, regarded the vaccination as a social responsibility, and had positive attitudes toward health authorities’ regular briefings were more likely to repeat the vaccination. In contrast, those who directly or indirectly experienced COVID-19 vaccine side effects and who showed psychological reactance against the government’s vaccination recommendation were less likely to repeat the vaccination. Our research indicates that empathetic communication, promotion of the prosocial aspect of vaccination, and regular and transparent provision of vaccine information are essential for promoting the intention to repeat the COVID-19 vaccination.

## 1. Introduction

The coronavirus disease 2019 (COVID-19) has tremendously affected people’s lives and livelihoods since the first case was reported in December 2019. As of 17 February 2023, more than 750 million people worldwide have been infected with the disease, including 6.84 million deaths [1]. Even though nonpharmaceutical interventions were effective in limiting the spread of the disease, they entailed societal costs, including economic burden and health problems [2]. Vaccines were seen as essential to getting back to ‘normal’. Pharmaceutical companies, such as AstraZeneca, Moderna, and Pfizer-BioNTech, jumped into the development of vaccines, and COVID-19 vaccines were developed at unprecedented speeds [3]. In a survey of 15 countries conducted in January 2021, 11 of 15 countries showed high levels of intent (more than 65% of respondents agreed to get vaccinated) to take a COVID-19 vaccine, even though there were concerns about the vaccine adverse events and the pace of the clinical trials [4]. Research conducted prior to the Omicron wave revealed that factors, such as demographic factors, beliefs on infection and vaccination, health literacy and governments’ policies (e.g., vaccine approval, vaccination mandate policies, and free vaccination program), influenced COVID-19 vaccination intention and inoculation [5,6,7,8,9]. As of February 2023, over 5.54 billion people worldwide have received at least one dose of a COVID-19 vaccine, and vaccination has played a role in preventing COVID-19-related hospitalization and death [10,11].

The severity of COVID-19 declined with the emergence of the Omicron variant and hybrid immunity, which is acquired from both prior SARS-CoV-2 infection and vaccination [12,13]. Many countries, including South Korea, scrapped or eased COVID-19 restrictions like mask-wearing and physical distancing in 2022, believing the coronavirus was no longer as fatal as it used to be [14]. However, the COVID-19 pandemic is not over, and public health experts recommend getting COVID-19 vaccines every year, similar to the annual flu shot [10,15]. As the positive outcomes of vaccination are expected only when individuals are willing to repeat the immunization, maintaining a high vaccination intention remains a public health challenge. In a survey of 23 countries, for instance, COVID-19 vaccine hesitancy increased between 2021 and 2022 in eight countries, including the United Kingdom, Mexico, and South Africa (ranging from 1.0% to 21.1%) [16]. In the same study, from 1.1% to 28.9% of those who had been vaccinated were hesitant to receive booster vaccines (on average 12.1%) [16].

**Figure 1:**
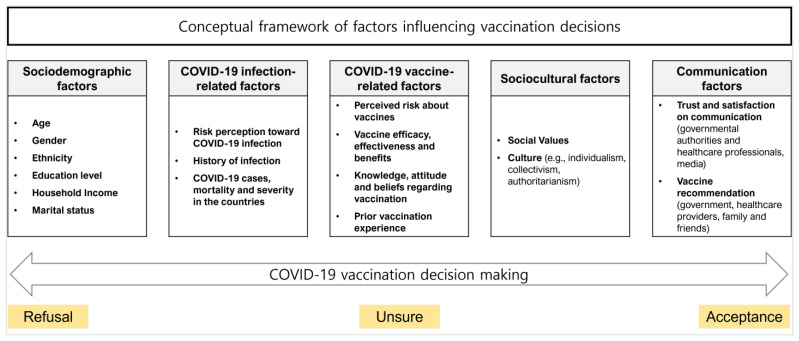
Conceptual framework of vaccination-related factors (adapted with permission from Ref. [19]. 2021, Joshi, Kaur, Kaur, Grover, Nash, and El-Mohandes).

The vaccination decision-making process is complex and multifaceted; factors at different levels, from the micro (e.g., knowledge, risk perception, and attitude) to the macro (e.g., public policy and trust in government and health authorities) levels, are interrelated and affect the decision-making process [17,18]. Joshi et al. [19] suggested a conceptual framework of factors influencing vaccination decisions and categorized the factors into four groups: (1) sociodemographic factors, (2) COVID-19 infection-related factors, (3) COVID-19 vaccine-related factors, and (4) communication factors. In addition to these four categories, sociocultural factors, such as religious and cultural norms, and social trust are known to influence vaccination decision-making [20,21]. However, most studies exploring the factors predicting intention to receive COVID-19 booster shots have focused on sociodemographic factors and investigated individuals’ willingness to receive a single booster shot [22]. In this study, sociocultural factors were added to the four main factors presented by Joshi et al. [19] to investigate the factors that affect the intention to repeat the COVID-19 vaccination and to suggest communication strategies for a sustainable COVID-19 vaccination program (Figure 1).

## 2. Methods

### 2.1. Participants and Procedures

We conducted an online survey from 18 January to 26 January 2022. This is when the Omicron, which is more transmissible than the original SARS-CoV-2 virus and the Delta variant, became the dominant variant, increasing the number of daily new cases in Korea [23]. The Korean government adopted a vaccine pass with an expiration date of 6 months, and people were required to get a booster shot due to the validity period [24]. The participants, adults aged 18 years and over living in Gyeonggi-do, were recruited using the survey panel of Hankook Research Company, one of the major research companies in South Korea. Samples were extracted by sex, age, and eight regions in Gyeonggi-do using proportional and quota sampling methods. The survey Uniform Resource Locator (URL) explaining the purpose of the research was sent via text message and email. The study was approved by the Institutional Review Board of Seoul National University (IRB No. 2201/002-008). The survey was completed by a total of 1000 participants, each of whom had previously consented to participate in the study.

### 2.2. Measures

#### 2.2.1. Dependent Variable

We asked participants whether they would get vaccinated (or receive booster shots for those who had already been vaccinated at least once) against COVID-19. Response options included “definitely not”, “probably not”, “probably would”, “definitely would”, and “unsure”. For the analysis, positive responses (definitely/probably would) were classified as ‘acceptance’, and negative (definitely/probably not) and ‘unsure’ responses were classified as ‘hesitant/unsure’ [25].

#### 2.2.2. Independent Variables

Sociodemographic factors included sex, age, education level, marital status, monthly household income, and health status (chronic disease), referring to previous research [19,22]. We inquired participants if they had any chronic diseases (e.g., high blood pressure, stroke, diabetes) that had been diagnosed or being treated by a doctor over the past year in order to assess their health status, referring to the Korean National Health and Nutrition Examination Survey (KNHANES) [26].

The COVID-19 infection and related factors included risk perception and history of infection. We measured the risk perception of COVID-19 infection with two items: perceived likelihood and perceived severity [27]. Participants were asked to assess the probability of being harmed by the COVID-19 infection (or reinfection) and the extent of harm that such infection (or reinfection) would cause, using a 5-point Likert-type scale (1 = “very unlikely” to 5 = “very likely”, and 1 = “never serious” to 5 = “very serious”, respectively). According to a prior study, the level of risk perception was determined by averaging the two items [28]. For a history of infection, we asked participants if they or anyone close to them (e.g., family, friends, and colleagues) had been infected with COVID-19.

COVID-19 vaccine-related factors included two variables regarding vaccination experiences: COVID-19 vaccination status and experience of vaccine adverse events. Participants were asked to report their vaccination status: “did not take”, “got a first dose”, “completed primary series (two doses with AstraZeneca, Pfizer-BioNTech, and Moderna, or one dose with Janssen)”, and “got a third shot (booster shot)”. We also asked them if they or anyone close to them experienced adverse reactions after receiving the COVID-19 vaccination.

Sociocultural factors included three variables: political ideology, authoritarian attitude, and collective responsibility. Political ideology was measured in a single item (0 liberal–10 conservatives, 11 unsure) and divided into four categories for analysis: ‘liberal (0–3)’, ‘moderate (4–6)’, ‘conservative (7–10)’, and ‘unsure’. Authoritarian attitudes were assessed with three items developed by the authors based on previous studies [29,30,31]. Examples include: “In response to the COVID-19 pandemic, questioning about or disregarding the authority of scientific knowledge and technologies represents a risk”. The degree of agreement with the statements was measured on a 5-point Likert-type scale (1 = “strongly disagree” to 5 = “strongly agree”), and the mean of the three items was calculated. Cronbach’s alpha value of the items was 0.749, indicating an acceptable internal reliability level. A question from the KFF’s poll on the COVID-19 vaccine was used to assess collective responsibility [32]. Respondents were asked to choose the option that best represented their perspective: “Getting vaccinated against COVID-19 is a personal choice to prevent the spread of the coronavirus”, “Getting vaccinated is everyone’s responsibility to protect the health of others”, “Both”, “Neither”, and “Don’t know”.

For communication factors, we measured the attitude toward the regular briefing of health authorities with three items and averaged them [33]. Respondents were asked to rate the reliability and helpfulness of the regular briefings, as well as how much attention they paid to the briefings using a 5-point Likert-type scale (1 = “not at all” to 5 = “very much”). Internal reliability of the three items was good (Cronbach’s alpha = 0.890). Additionally, the psychological reactance against the government’s vaccination recommendation was estimated using five items developed by the authors with reference to a prior study [34]. The examples include “The health authorities seem to force vaccination” and “The health authorities do not seem to fully understand the public’s doubts and concerns about vaccines”. Participants were asked to indicate the extent to which they agreed with the statements using a 7-point Likert-type scale (1 = “strongly disagree” to 7 = “strongly agree”), and the mean of the five items was calculated. Cronbach’s alpha value of the items was 0.956, which indicates an excellent internal reliability level. The ordinal Likert-type scales were treated as continuous scales in bivariate analysis and logistic regression analysis.

### 2.3. Data Analysis

A descriptive analysis was conducted to provide information on the sociodemographic characteristics of the survey participants. Quantitative information was presented using percentages (%) or means (M) and standard deviations (SD). The *t*-test and the chi-squared test were used to compare differences between the vaccine-hesitant/unsure group and the vaccine-acceptance group.

A hierarchical logistic regression analysis was performed to identify the factors that affect the intention to repeat COVID-19 vaccination, comparing nested regression models. We present five models: (1) Model 1: A model with sociodemographic factors; (2) Model 2: COVID-19 infection-related factors are added into the model; (3) Model 3: COVID-19 vaccine-related factors were entered into the model; (4) Model 4: sociocultural factors were entered into the model; and (5) Model 5: communication factors were added into the model. All analyses were performed using R version 4.1.1 (R Foundation for Statistical Computing, Vienna, Austria).

## 3. Results

### 3.1. Characteristics of Participants

Of a total of 1000 participants, 50.5% were male and 49.5% were female. The average age of the participants was 46.2 years old (SD = 14.8), and more than three-quarters had received some higher education (76.9%). A total of 60.3% of participants were married, and 28.0% had a monthly household income of 3.00–4.99 million KRW, followed by more than 7.00 million KRW (26.8%), 5.00–6.99 million KRW (24.9%), and less than 3.00 million KRW (20.3%). Approximately half of the participants (53.1%) had been diagnosed with chronic diseases (Table 1).

### 3.2. Differences in Variables That Affect Intention to Repeat COVID-19 Vaccination

Table 1 presents the results of the *t*-test and chi-squared test. People who were willing to repeat COVID-19 vaccination (78.9%) outnumbered those who were hesitant/unsure (21.1%). There were some differences in demographic factors between the two groups. Approximately half of the acceptance group (48.3%) was 50 years old and older, while approximately half (48.3%) of the hesitant/unsure group was under 40 years old (*p* < 0.001). When compared with the hesitant/unsure group, more people in the acceptance group were married (50.2% vs. 63.0%) and had chronic diseases (38.4% vs. 49.2%) (*p* < 0.01).

There were significant differences between the two groups in COVID-19 infection-related factors, COVID-19 vaccine-related factors, sociocultural factors, and communication factors, except for a history of infection. The accepting group had higher risk perceptions (*Mean* = 3.14) than the hesitant/unsure group (*Mean* = 2.91) (*p* < 0.001). Most participants in the acceptance group have been vaccinated against COVID-19 at least once (97.7%), whereas nearly a quarter (24.6%) of those in the hesitant/unsure group had not been vaccinated (*p* < 0.001). COVID-19 vaccine adverse events were affiliated with 72.5% of the hesitant/unsure group and 59.1% of the acceptance group (*p* < 0.001). There were 26.1% of politically conservative people in the hesitant/unsure group and 17.0% in the acceptance group (*p* < 0.001). The authoritarian attitude was higher in the vaccine acceptance group (*Mean* = 3.60) than in the hesitant/unsure group (*Mean* = 3.09) (*p* < 0.001). More than half of the hesitant/unsure participants (56.4%) considered vaccination as a personal choice, while nearly half of the accepting participants (48.3%) considered vaccination as a social responsibility. Members of the acceptance group had a more favorable attitude toward the regular briefings and lower psychological reactance to the government’s recommendation for vaccination than those in the hesitant/unsure group (*p* < 0.001).

### 3.3. Factors Associated with Intention to Repeat COVID-19 Vaccination

Table 2 presents the outcomes of a multivariable hierarchical logistic regression analysis. In Model 1, which includes sociodemographic factors, age was the sole predictor of the intention to repeat COVID-19 vaccination (*R*^2^ = 0.028). A one-year increase in age was related to a significant increase in the probability of repeating the COVID-19 vaccination (OR = 1.02, 95% CI = 1.01–1.03). In Model 2, which contained variables related to COVID-19 infection, risk perception was a significant predictor of compliance with repeat COVID-19 vaccinations (OR = 1.63; CI = 1.29–2.06). In Model 3, a model in which COVID-19 vaccine-related factors were included, people who had been vaccinated at least once (OR_1–2_ = 8.82; CI_1–2_ = 4.91–16.51) and who had received a booster shot (OR_3_ = 18.91; CI_3_ = 10.34–35.98) were more willing to repeat the vaccination compared with unvaccinated people. Those who had vaccine adverse events were less likely to be vaccinated again (OR = 0.48; CI = 0.33–0.69). In Model 4, a model with sociocultural factors, those who had a stronger authoritarian attitude were more likely to repeat COVID-19 vaccinations (OR = 1.60; CI = 1.22–2.10). Compared to those who considered vaccination as a personal decision, those who considered it as a social responsibility (OR = 8.44; CI = 5.00–14.58) and those who considered it as both a personal choice and a social responsibility (OR = 4.23; CI = 2.71–6.66) were more likely to receive an additional vaccine shot. In Model 5, which includes communication factors, those with a positive attitude toward regular briefings were more likely to be vaccinated again (OR = 1.56; CI = 1.20–2.03). People with higher psychological reactance to the government’s vaccination recommendation were less likely to accept repeat COVID-19 vaccinations (OR = 0.74; CI = 0.63–0.86). After controlling for other variables, vaccination status (OR_1–2_ = 5.13; CI_1–2_ = 2.61–10.42; OR_3_ = 6.29; CI_3_ = 3.10–13.09) and vaccine adverse events (OR = 0.53; CI = 0.34–0.82) were significant predictors of intention to repeat COVID-19 vaccination. 

## 4. Discussion

Immunity gained through vaccination and previous infection, as well as the characteristics of the Omicron variant (the infection rate is faster but less severe than existing viruses), mitigated the severity of COVID-19 and risk perception [13,35,36]. As governments decided to live with and manage the coronavirus, they relaxed or lifted restrictions and vaccination remains the most essential way for health protection [37]. Prior research demonstrated that COVID-19 vaccination, especially for groups at high risk of infection, was more cost-effective than no vaccination [38].

South Korea is regarded as an exemplary case of a COVID-19 vaccination program in the OECD, with a high vaccination rate (87.0%) [9,11]. Despite its successful vaccination program before the Omicron wave, making the public repeat the COVID-19 vaccination is still a challenge. In a study of Canadian adults, for example, acceptance of annual shots was lower than acceptance of a booster shot (third dose), suggesting that vaccine uptake would be dismal if citizens are required to receive COVID-19 vaccination annually [39]. The reported willingness to receive booster shots in other countries varied from 19.3% to 82.4% [39,40,41,42]. In this study, 78.9% of Korean citizens were willing to repeat the COVID-19 vaccination. We explored five factors that influence this intention, and several notable findings could provide insights into vaccine communication for stainable COVID-19 vaccination programs.

First and foremost, most sociodemographic factors, which were the focus of previous studies, were not significant predictors of the intention to repeat the COVID-19 vaccination [22]. Instead, the majority of variables related to COVID-19 infection and vaccination, as well as socio-cultural and communication variables, were associated with the willingness to repeat the COVID-19 vaccination. This indicates that psychological, experiential, and contextual factors, in addition to sociodemographic factors, should be considered to boost the motivation to repeat the vaccination [22,43,44]. In this study, participants who perceived themselves to be more susceptible to contracting a disease or who perceived the severity of infection-related effects were more likely to accept repeated COVID-19 vaccinations, which was consistent with previous studies [19,27]. This can be explained by people’s desire to avoid negative health outcomes and motivation to adopt the action that is expected to reduce the likelihood or severity of harm [45]. It is essential to increase the perceived risk of infection, particularly among those who are physically vulnerable, in order to improve the willingness to repeat immunization [46]. This should be done along with messages about the benefits of vaccination, because loss-framed messages may elicit freedom threats and, consequently, psychological reactance from message recipients [47,48].

People who had a positive attitude toward regular briefing of health authorities were more likely to repeat the vaccination. Open and transparent communication is essential for building trust in health authorities, which impacts trust in vaccination [49,50]. Since the COVID-19 outbreak, the Korea Disease Control and Prevention Agency (KDCA) has regularly provided information about COVID-19, including statistical information (e.g., vaccination rates) and prevention guidelines (e.g., vaccination plans) [51]. Regular briefings have been an essential channel for the public to obtain trustful vaccination information, and vaccine communication may lead to acceptance of repeat vaccination [51,52]. Yet, changes in vaccine communication are needed. In this study, individuals who directly or indirectly experienced adverse events following immunization and who had psychological reactance against the government’s vaccination recommendation were less likely to repeat vaccination. People who had vaccination side effects are more likely to regret their previous vaccination decisions, resulting in decreased intention to get an additional dose [53]. As vague communication concealing undesirable vaccine features (e.g., vaccine side effects) has negative effects in the long run by eroding trust in health authorities, health authorities should keep communication about negative information as well as positive information regarding vaccination [54]. Additionally, messages using freedom-threatening language increase perceived threat and psychological reactance, which in turn decreased perceived message persuasiveness [48,55]. Therefore, health authorities need to be aware of the public’s concerns about vaccination adverse events and communicate with empathy [56]. This can be done, for example, by discussing the difficulties of those affected by vaccine side effects [57].

In the current study, people who received the COVID-19 vaccine at least once were more willing to repeat vaccination. Those who were vaccinated in the past might find vaccination useful and be more willing to accept vaccination in the future [58]. Sharing positive personal experiences with vaccination and reasons for vaccination can influence others’ willingness to vaccinate [59]. In addition, collective responsibility and individual ideology were related to vaccination intention in this study. Those who believed that vaccination is everyone’s responsibility for the health of society and those with an authoritarian attitude were more likely to receive repeat vaccinations. This finding parallels that of a previous study performed in Germany, Poland, and the United Kingdom, which indicated that people who favor obedience to authorities and established norms and who favor punishing the violation of social norms were more likely to receive the COVID-19 vaccine [60]. Therefore, vaccine uptake can be increased by promoting the prosocial aspect of vaccinations by raising people’s knowledge of herd immunity and eliciting empathy and sympathy for those who can benefit from it [61].

We recognize some limitations in our study. As it is a cross-sectional study, it is difficult to infer the causal relationship between dependent and independent variables. In addition, as the intention to repeat COVID-19 vaccination and the related factors may be changed depending on the pandemic context, the relationship between those variables can also change. The results of the current study may not fully reflect Korean citizens’ intention to repeat the COVID-19 vaccination following the survey. Despite these limitations, this study on the intention to repeat the COVID-19 vaccination suggests that psychological, experiential, and sociocultural factors should be considered in vaccination programs and that changes in vaccine communication are needed. These results may provide significant insights into how to deal with COVID-19 and other emerging infectious diseases in the future.

## 5. Conclusions

In response to the COVID-19 pandemic, vaccination has played a vital role in protecting citizens’ health and lives. The current study identified that COVID-19 infection and vaccine-related factors (risk perception, COVID-19 vaccination status, experience of adverse events), sociocultural factors (authoritarian attitude, collective responsibility), and communication factors (attitude on regular briefing, psychological reactance against vaccination recommendation) affect the intention to repeat the COVID-19 vaccination. The results suggest that effective vaccine communication is necessary for COVID-19 vaccination programs. This includes providing vaccine-related information regularly and transparently, being sensitive to people’s emotions, and encouraging prosocial vaccination.

## Figures and Tables

**Table 1:** Characteristics of participants by intention to repeat COVID-19 vaccination.

			Intention to Repeat COVID-19 Vaccination				
	All Subjects		Hesitant/Unsure		Acceptance		*t*-Test/Χ^2^
	N	%	N	%	N	%	*p*-Value
	1000	100.0	211	100	789	100	
**Sex**							0.750
Male	505	50.5	104	49.3	401	50.8	
Female	495	49.5	107	50.7	388	49.2	
**Age** (***M***(**SD**))	46.2 (14.8)		42.1 (15.4)		47.3 (14.4)		<0.001 ***
18–29 years	182	18.2	58	27.5	124	15.7	
30–39 years	165	16.5	44	20.8	121	15.3	
40–49 years	199	19.9	36	17.1	163	20.7	
50–59 years	201	20.1	30	14.2	171	21.7	
≥60 years	253	25.3	43	20.4	210	26.6	
**Education**							0.290
Less than High school	231	23.1	55	26.1	176	22.3	
College and above	769	76.9	156	73.9	613	77.7	
**Marital Status**							0.001 **
Single/Divorced/Widowed	397	39.7	105	49.8	292	37.0	
Married	603	60.3	106	50.2	497	63.0	
**Monthly Household Income** ^†^							0.214
<3.00	203	20.3	49	23.2	154	19.5	
3.00–4.99	280	28.0	48	22.8	232	29.4	
5.00–6.99	249	24.9	58	27.5	191	24.2	
≥7.00	268	26.8	56	26.5	212	26.9	
**Health status** (**Chronic Disease**)							0.007 **
No	531	53.1	130	61.6	401	50.8	
Yes	469	46.9	81	38.4	388	49.2	
**Risk Perception** (***M***(**SD**))	3.09 (0.64)		2.91 (0.74)		3.14 (0.60)		<0.001 ***
**History of Infection**							0.935
No	692	69.2	147	69.7	545	69.1	
Yes	308	30.8	64	30.3	244	30.9	
**Vaccination Status**							<0.001 ***
0	70	7.0	52	24.6	18	2.3	
1–2	402	40.2	70	46.0	305	38.7	
3 (booster shot)	52	52.8	62	29.4	466	59.0	
**Vaccine adverse events**							<0.001 ***
No	381	38.1	58	27.5	323	40.9	
Yes	619	61.9	153	72.5	466	59.1	
**Political ideology**							<0.001 ***
Conservative	189	18.9	55	26.1	134	17.0	
Moderate	584	58.4	122	57.8	462	58.5	
Liberal	190	19.0	23	10.9	167	21.2	
Don’t know	37	3.7	11	5.2	26	3.3	
**Authoritarian attitude** (***M*** (**SD**))	3.49 (0.73)		3.09 (0.82)		3.60 (0.66)		<0.001 ***
**Collective responsibility**							<0.001 ***
Personal Choice	213	21.3	119	56.4	94	11.9	
Everyone’s responsibility	408	40.8	27	12.8	381	48.3	
Both	349	34.9	53	25.1	296	37.5	
Neither	9	0.9	5	2.4	4	0.5	
Don’t know	21	2.1	7	3.3	14	1.8	
**Attitude on regular briefing** (***M*** (**SD**))	3.22 (0.89)		2.58 (0.91)		3.39 (0.80)		<0.001 ***
**Psychological reactance against the government’s vaccination recommendation** (***M*** (**SD**))	4.71 (1.58)		5.79 (1.44)		4.42 (1.49)		<0.001 ***

Notes. * ***p*** < 0.050, ** ***p*** < 0.010, *** ***p*** < 0.001. **^†^** million KRW (KRW 1 = USD 0.00076).

**Table 2:** Hierarchical logistic regression analysis of intention to repeat COVID-19 vaccination.

*Predictors*	Model 1	Model 2	Model 3	Model 4	Model 5
	OR (95% CI)	OR (95% CI)	OR (95% CI)	OR (95% CI)	OR (95% CI)
**Sex**					
Male	Ref	Ref	Ref	Ref	Ref
Female	0.94 (0.69–1.28)	0.87 (0.63–1.20)	1.03 (0.73–1.45)	0.91 (0.62–1.34)	0.89 (0.60–1.31)
**Age**	1.02 (1.01–1.03) **	1.02 (1.01–1.03) **	1.00 (0.99–1.02)	1.00 (0.98–1.01)	1.00 (0.98–1.01)
**Education**					
Less than High school	Ref	Ref	Ref	Ref	Ref
College and above	1.43 (0.99–2.06)	1.42 (0.98–2.06)	1.29 (0.85–1.93)	1.28 (0.80–2.02)	1.32 (0.82–2.11)
**Marital Status**					
Single/Divorced/Widowed	Ref	Ref	Ref	Ref	Ref
Married	1.22 (0.83–1.78)	1.18 (0.80–1.74)	1.44 (0.95–2.18)	1.5 (0.95–2.38)	1.6 (0.99–2.56)
**Monthly Household Income**	1.00 (0.94–1.07)	1.00 (0.94–1.07)	0.99 (0.92–1.07)	0.98 (0.90–1.06)	0.98 (0.90–1.06)
**Health status** (**Chronic Disease**)					
No	Ref	Ref	Ref	Ref	Ref
Yes	1.29 (0.93–1.81)	1.24 (0.88–1.73)	1.37 (0.95–1.99)	1.37 (0.91–2.08)	1.41 (0.92–2.17)
**Risk perception**		1.63 (1.29–2.06) ***	1.61 (1.24–2.09) ***	1.33 (1.01–1.77) *	1.28 (0.95–1.71)
**History of Infection**					
No		Ref	Ref	Ref	Ref
Yes		1.00 (0.72–1.42)	1.05 (0.73–1.52)	1.04 (0.70–1.56)	1.11 (0.73–1.69)
**Vaccination status**					
0			Ref	Ref	Ref
1–2			8.82 (4.91–16.51) ***	5.51 (2.85–11.01) ***	5.13 (2.61–10.42) ***
3			18.91 (10.34–35.98) ***	7.35 (3.69–15.04) ***	6.29 (3.10–13.09) ***
**Vaccine adverse events**					
No			Ref	Ref	Ref
Yes			0.48 (0.33–0.69) ***	0.51 (0.33–0.77) **	0.53 (0.34–0.82) **
**Political ideology**					
Conservative				Ref	Ref
Moderate				1.44 (0.89–2.30)	1.27 (0.78–2.06)
Liberal				1.76 (0.92–3.43)	1.17 (0.59–2.36)
Don’t know				0.77 (0.31–1.97)	0.63 (0.25–1.68)
**Authoritarian attitude**				1.60 (1.22–2.10) **	1.22 (0.91–1.65)
**Collective responsibility**					
Personal choice				Ref	Ref
Everyone’s responsibility				8.44 (5.00–14.58) ***	4.83 (2.75–8.61) ***
Both				4.23 (2.71–6.66) ***	3.08 (1.92–4.96) ***
Neither				1.30 (0.25–6.66)	1.18 (0.23–5.91)
Don’t know				1.71 (0.63–5.01)	1.40 (0.50–4.16)
**Attitude on regular briefing**					1.56 (1.20–2.03) **
**Psychological reactance against the government’s vaccination recommendation**					0.74 (0.63–0.86) ***
***R*^2^ Tjur**	0.028	0.047	0.186	0.311	0.346
**Δ** ***R* ^2^**		0.019 ***	0.139 ***	0.125 ***	0.035 ***

Note. * *p* < 0.050, ** *p* < 0.010, *** *p* < 0.001.

## Data Availability

The data used and analyzed in this study are available from the corresponding author upon reasonable request.
